# Combination of *Lactobacillus fermentum* NS9 and aronia anthocyanidin extract alleviates sodium iodate-induced retina degeneration

**DOI:** 10.1038/s41598-023-34219-3

**Published:** 2023-05-24

**Authors:** Yan Xing, Shan Liang, Limei Zhang, He Ni, Xueqin Zhang, Jiancheng Wang, Liu Yang, Shuangshuang Song, Hai-Hang Li, Chenxi Jia, Feng Jin

**Affiliations:** 1 Guangdong Provincial Key Lab of Biotechnology for Plant Development, School of Life Sciences, South China Normal University, Guangzhou, 510631 China; 2 Research Laboratory of Antioxidation & Anti-Aging, Guozhen Health Technology (Beijing) Co., Ltd., Beijing, 102206 China; 3 Key Laboratory of Microbial Physiological and Metabolic Engineering, Institute of Microbiology, Chinese Academy of Sciences, Beijing, 100101 China; 4 State Key Laboratory of Proteomics, Beijing Proteome Research Center, Institute of Lifeomics, National Center for Protein Sciences (The PHOENIX Center), Beijing, 102206 China; 5 Key Laboratory of Mental Health, Institute of Psychology, Chinese Academy of Sciences, Beijing, 100101 China

**Keywords:** Microbiology, Diseases, Health care

## Abstract

It is important to explore the effective approaches to prevent dry age-related macular degeneration (AMD). In this study, significantly decreased full-field electroretinograms wave amplitudes and disordered retina structures were detected in rat retinas of sodium iodate induced dry AMD model. Six a- and b-wave amplitudes and the antioxidant activities were significantly increased, and the outer nuclear layer thickness was significantly improved in the rat retinas treated with the combination of *Lactobacillus fermentum* NS9 (LF) and aronia anthocyanidin extract (AAE) compared with the model. The effects were much better than the treatment with AAE alone. The proteomics analysis showed the expressions of α-, β- and γ-crystallins were increased by 3–8 folds in AAE treated alone and by 6–11 folds in AAE + LF treatment compared with the model, which was further confirmed by immuno-blotting analysis. Analysis of gut microbial composition indicated that higher abundance of the genus *Parasutterella* and species *P. excrementihominis* was found in the AAE + LF treatment compared with the other groups. The results indicated that the combined treatment of AAE + LF is a potential way to prevent the retina degeneration which is significantly better than the AAE treated alone.

## Introduction

Age-related macular degeneration (AMD) is known as a serious eye disease due to degenerative structural damage and loss of function of the retina which results in a progressive loss of central vision and blindness^1,2^. The dry AMD accounts for approximately 90% of the total AMD patients^1^. Currently, lack of effective treatments to manage the dry AMD is a major problem. It is necessary to explore possible therapeutic options for dry AMD.

As an age-related disorder, AMD is often associated with Alzheimer's disease (AD), in which visual abnormalities are prominent and are believed to develop before cognitive decline. The two diseases share several features, including deposits of β-amyloid (Aβ), chronic inflammation, and oxidative stress^3^. We have demonstrated ingestion of *Lactobacillus* NS strain reduced anxiety and improved cognitive function in the hyperammonemia rats^4^. *L. helveticus* NS8 and *L. fermentum* NS9 exhibited strain-specific effects for regulation of the brain peptidome^5^. *L. fermentum* NS9 also normalized the composition of gut microbiota and alleviated the ampicillin-induced impairment in memory retention^6^.

Though the exact mechanism of dry AMD remains unknown, oxidative stress-induced damage of retinal pigment epithelial cells (RPE) and photoreceptors is believed to be strongly implicated in AMD pathogenesis^7^. A recent study showed that *L. fermentum* alleviated the oxidative stress and inflammation in D-galactose-induced aging model^8^. Increasing studies also indicate that gut microbiota play great role in eye health and the gut-retina axis was involved in the age-related macular disorders^9^. Microbial dysbiosis could change the blood-retina barrier permeability and link with the retinal degeneration^10^. Gut microbiota could also act as regulatory factors in the inflammation and immune responses^11^. It has been reported that *L. paracasei* KW3110 suppressed age-related chronic inflammation and retinal ganglion cell (RGC) loss through modulation of gut microbiota composition and immune system function^12^.

In previous study, we found aronia anthocyanidin had protective effects on rat retina, with significant increased expression of crystallin proteins, while the effects on electroretinogram (ERG) and rat retina structure were modest^13^. There are mutual interactions between polyphenols and the gut microbiota, and increased efficacy has been observed when probiotics combined with prebiotics^14,15^.

Sodium iodate (NaIO_3_) is an oxidative agent, and it can induce selective RPE damage. The murine NaIO_3_ model has been widely used to study dry AMD since it results in reproducible, patchy retinal degeneration^16–18^.In this study, we investigated the effects and possible mechanisms of aronia anthocyanidin extract (AAE) together with *L. fermentum* NS9 (LF) on rat retina in the NaIO_3_ induced dry AMD model, in comparison with AAE treatment alone.

## Results

### Protective effects of the treatment on NaIO_3_-induced rat retina damages

**Figure 1:**
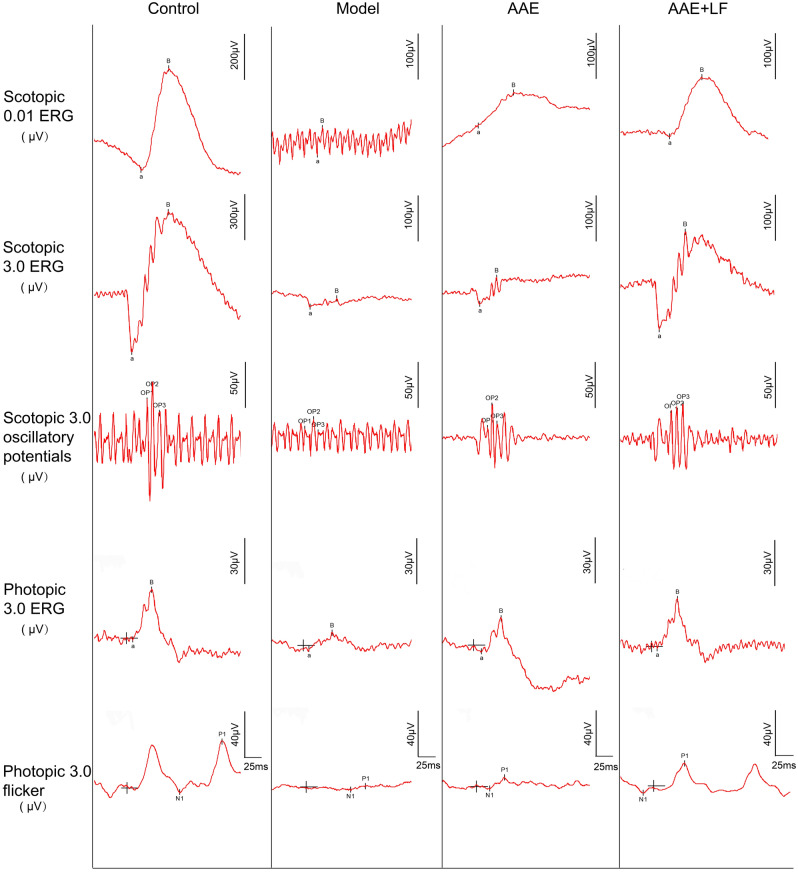
Recorded spectra of full-field ERG of the rat retinas in different treatments. Scotopic 0.01 ERG, Scotopic 3.0 ERG, Scotopic 3.0 oscillatory potentials, Photopic 3.0 ERG and Photopic 3.0 flicker of the rat retina were recorded according to the ISCEV (International Society of Clinical Electrophysilological Vision) standard. Control: control without treatment; Model: damage model by 30 mg/kg body weight NaIO_3_ tail vein injection; AAE: aronia anthocyanidin (60 mg/kg body weight) treatment of the damage model; AAE + LF: treatment with 60 mg/kg body weight aronia anthocyanidin and 10^8^ CFU/ml *L. fermentum* NS9 of the damage model.

**Figure 2:**
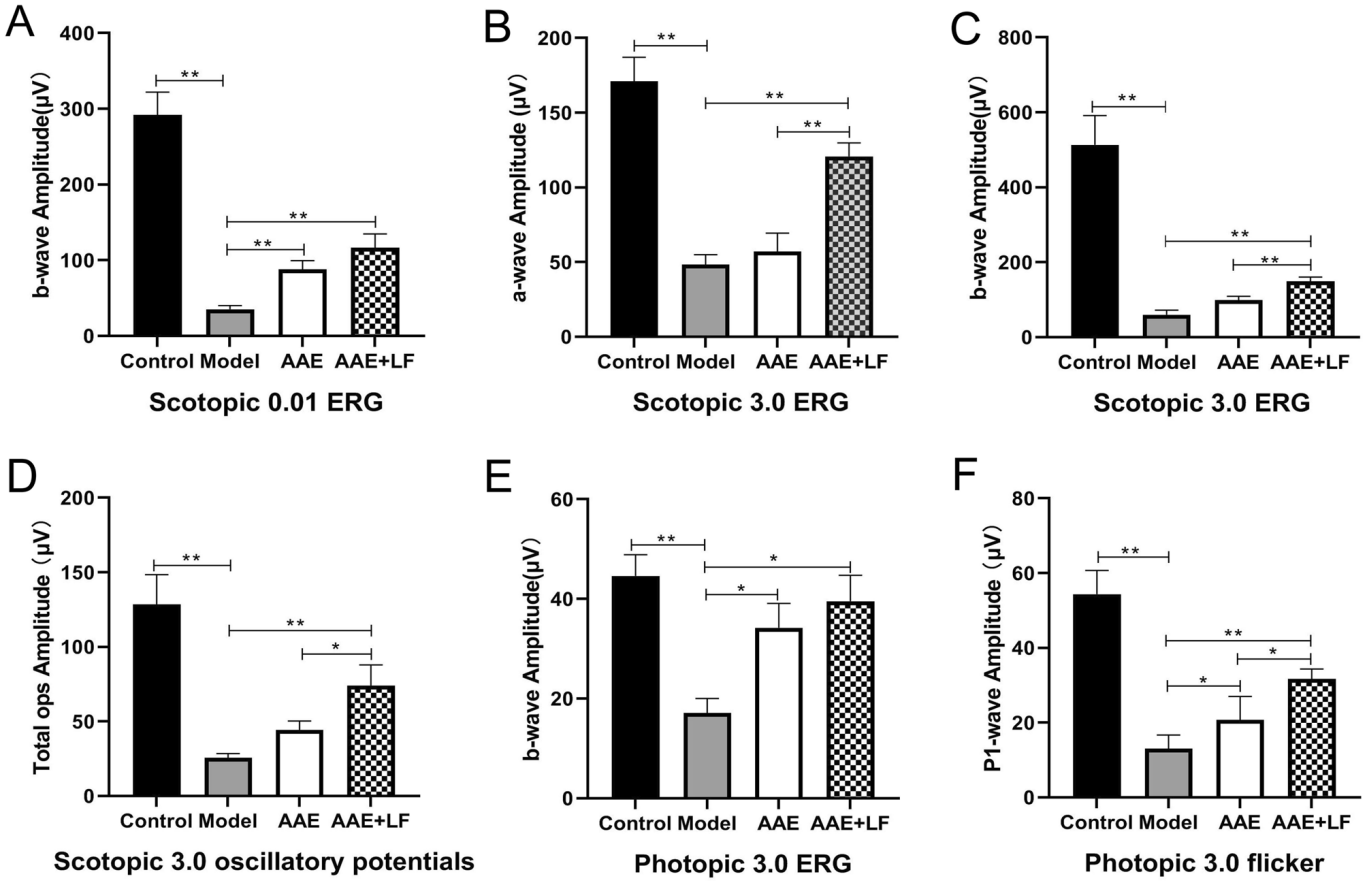
Average ERG amplitudes of the rat retinas in different treatments. (**A**) b-wave of Scotopic 0.01 ERG; (**B**, **C**) a- and b-wave of Scotopic 3.0 ERG; (**D**) total amplitude of Scotopic 3.0 oscillatory (3 ops); (**E**) b-wave of Photopic 3.0 ERG; (**F**) P1-wave amplitude of Photopic 3.0 flicker. Data shown are the mean ± standard deviation (n = 10). *p < 0.05, **p < 0.01 (one-way ANOVA followed by Tukey’s test).

ERG is a common and sensitive measurement to evaluate retinal function^19^. In Model group, ERG amplitudes were significantly decreased compared with the Control group, including the decreases of b-wave of Scotopic 0.01 ERG by 88.01%, a- and b-wave of Scotopic 3.0 ERG by 71.75% and 90.34%, total amplitude of Scotopic 3.0 oscillatory (3 ops) by 80.10%, b-wave of Photopic 3.0 ERG by 61.51% and P1-wave amplitude of Photopic 3.0 flicker by 76.01% respectively (Figs. 1 and 2A–F), which indicated a global worsening function of rat retina after NaIO_3_ treatment. Compared with the Model group, the AAE treatment significantly improved the b-wave amplitudes of Scotopic 0.01 ERG, Photopic 3.0 ERG and Photopic 3.0 flicker by 150.96%, 99.36% and 58.90% , respectively (Figs. 1 and 2A,E,F), which was coincident with our previous finding^13^. The protective effects were more significant in treatment of AAE together with LF. ERGs a- and b-wave amplitudes for all six different measurements were significantly increased by 233.35%, 149.90%, 201.43%, 189.55%, 130.42% and 142.79%, respectively, compared with the model (Figs. 1 and 2A–F). The Scotopic 3.0 ERG a- and b-wave was further increased by 112.07% and 50.28%, total amplitude of Scotopic 3.0 oscillatory by 66.73% and P1-wave amplitude of Photopic 3.0 flicker by 52.80% compared with AAE group (Figs. 1 and 2B–D,F).

**Figure 3:**
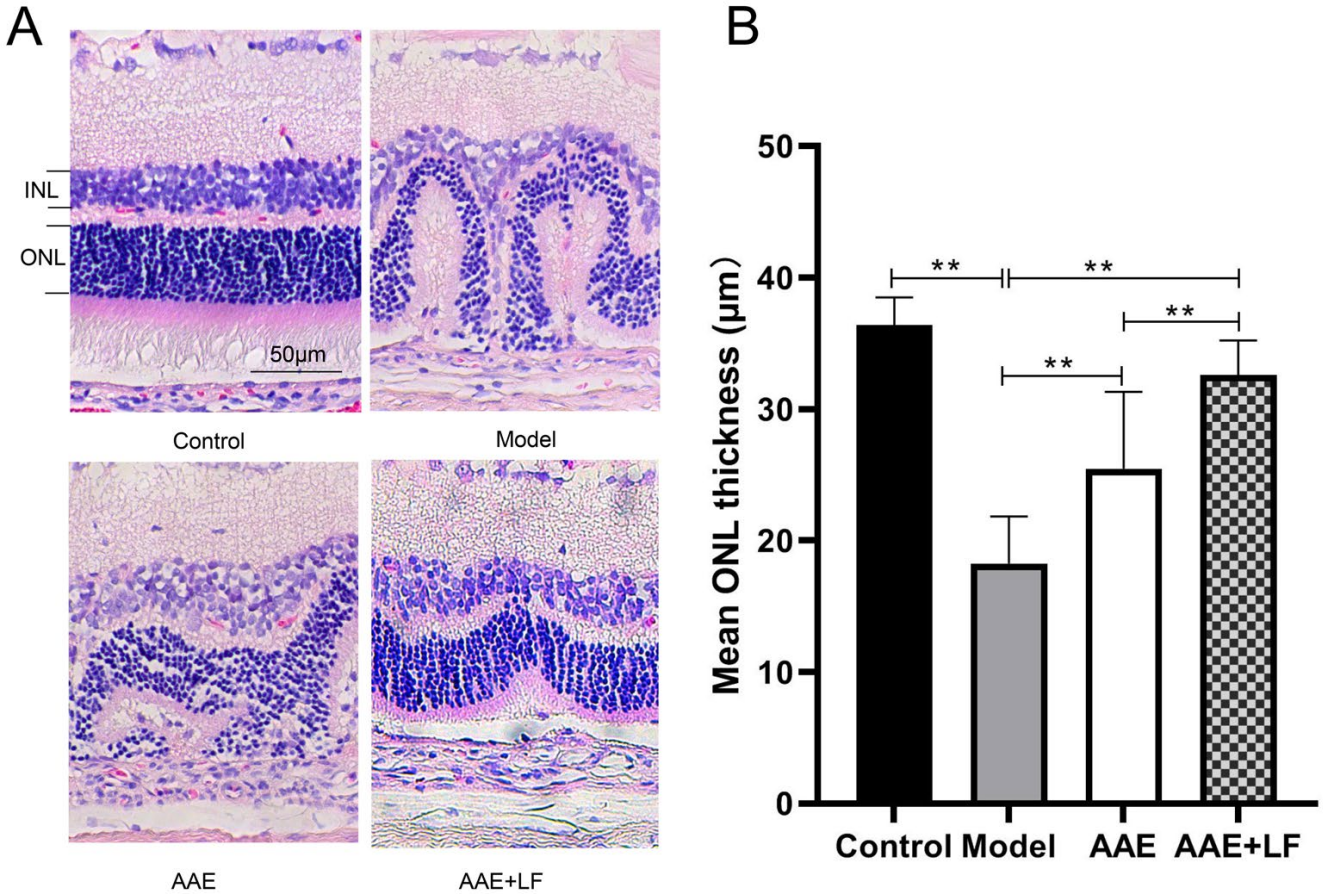
Protective effects of AAE alone and AAE + LF on rat retinal structure. (**A**) Images of H&E stained rat retina section, taken at 200 × magnification; (**B**) Outer nuclear layer thickness of the retinas, data shown are the mean ± standard deviations (n = 4). *p < 0.05, **p < 0.01 (one-way ANOVA followed by Tukey’s test). *ONL* outer nuclear layer, *INL* inner nuclear layer. Bar equals 50 µm.

The protective function of the treatments was also demonstrated in histological analysis of the retina structure. The retina in the NaIO_3_-induced damage model showed a disordered structure and a reduction of cell layers with messed outer and inner nuclear layers (ONL and INL). Retinas in AAE group showed an improvement on its structure and cell layers. The improvement was further significant in AAE + LF group, the alignment of their nucleus was relatively in order (Fig. 3A). The mean ONL thickness was significantly reduced by 49.89% in Model compared with the Control. Compared with the Model, the ONL thickness was significantly increased by 39.53% and 78.67% respectively in AAE and AAE + LF (Fig. 3B). The mean ONL thickness was increased by 28.06% in AAE + LF compared with the AAE group (Fig. 3B).

### Antioxidative effect of the treatment on the rat retinas

**Figure 4:**
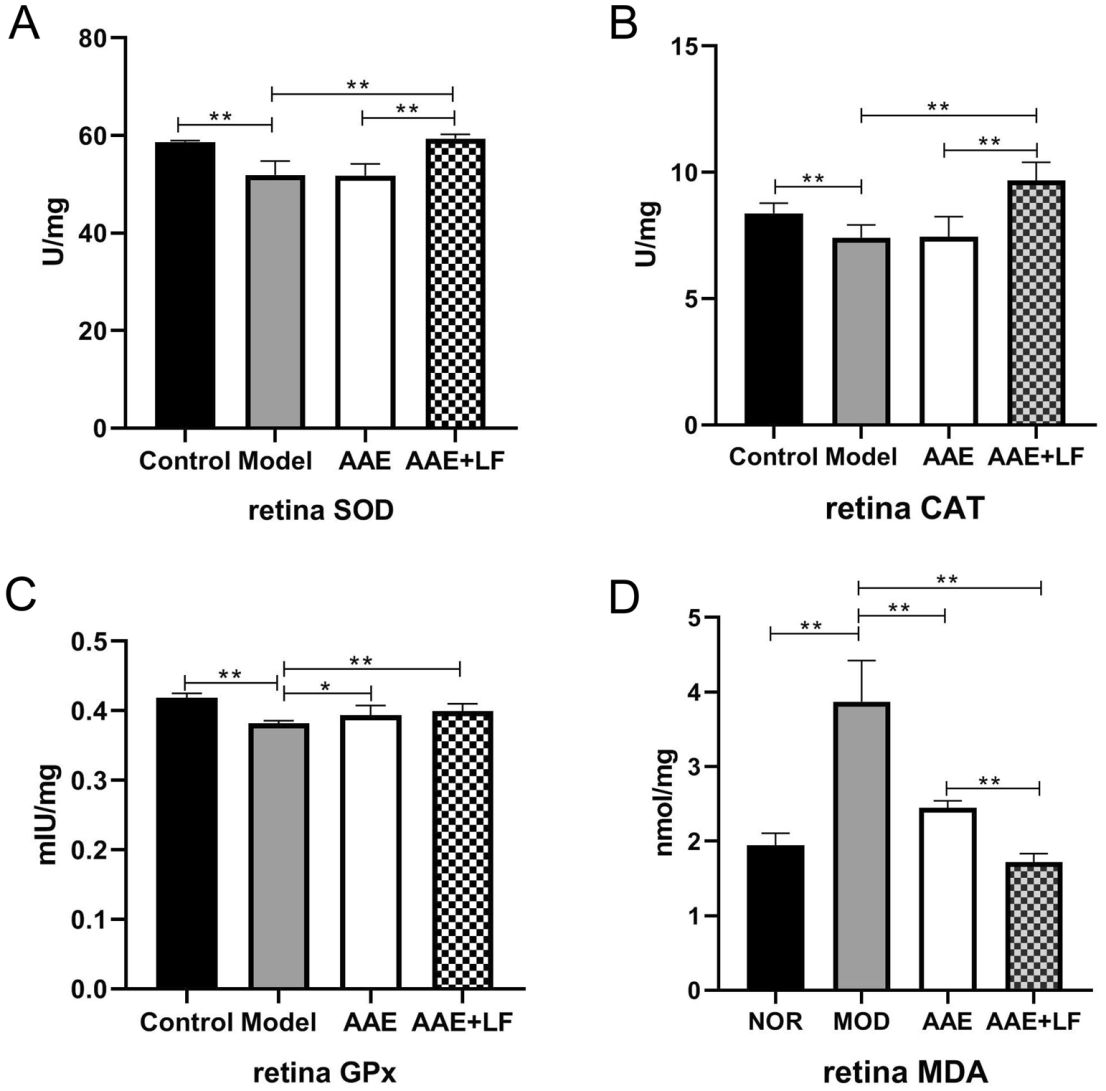
Antioxidant capacity of the rat retinas. (**A**–**C**) SOD, CAT and GPx activities, respectively; (**D**) MDA content. Data shown are the mean ± standard deviations (n = 8). *p < 0.05, **p < 0.01 (one-way ANOVA followed by Tukey’s test). *SOD* superoxide dismutase, *CAT* catalase, *GPx* glutathione peroxidase, *MDA* malondialdehyde.

As shown in Fig. 4, the activities of antioxidant enzymes superoxide dismutase (SOD), catalase (CAT), glutathione peroxidase (GPx) were reduced by 11.52%, 11.50% and 8.79%, respectively, and the malondialdehyde (MDA) level was increased by 99.26% in the Model group compared with those in the Control, which indicated a deterioration of antioxidant status in the damaged rats. Limited increase of GPx activity and decrease of MDA level were observed in the AAE treated rats (Fig. 4C,D). The AAE + LF treatment showed significant improvement on antioxidant capacity in the retinas. The enzyme activities of SOD, CAT and GPx were increased by 14.37%, 30.53% and 4.72%, respectively, and the MDA level was reduced by 55.57% in the AAE + LF treatment compared with the model (Fig. 4). The SOD, CAT activities were increased by 14.67% and 30.00%, and the MDA level was decreased by 29.88% in the AAE + LF compared with the AAE treatment (Fig. 4). The results suggest that the treatment of AAE + LF significantly increased the antioxidant capacity by upregulating antioxidant enzyme activities and decreasing the produce of MDA in the damaged rat retina.

### Effect of the treatments on the expression of crystallin proteins and caspase 3 in rat retina

**Figure 5:**
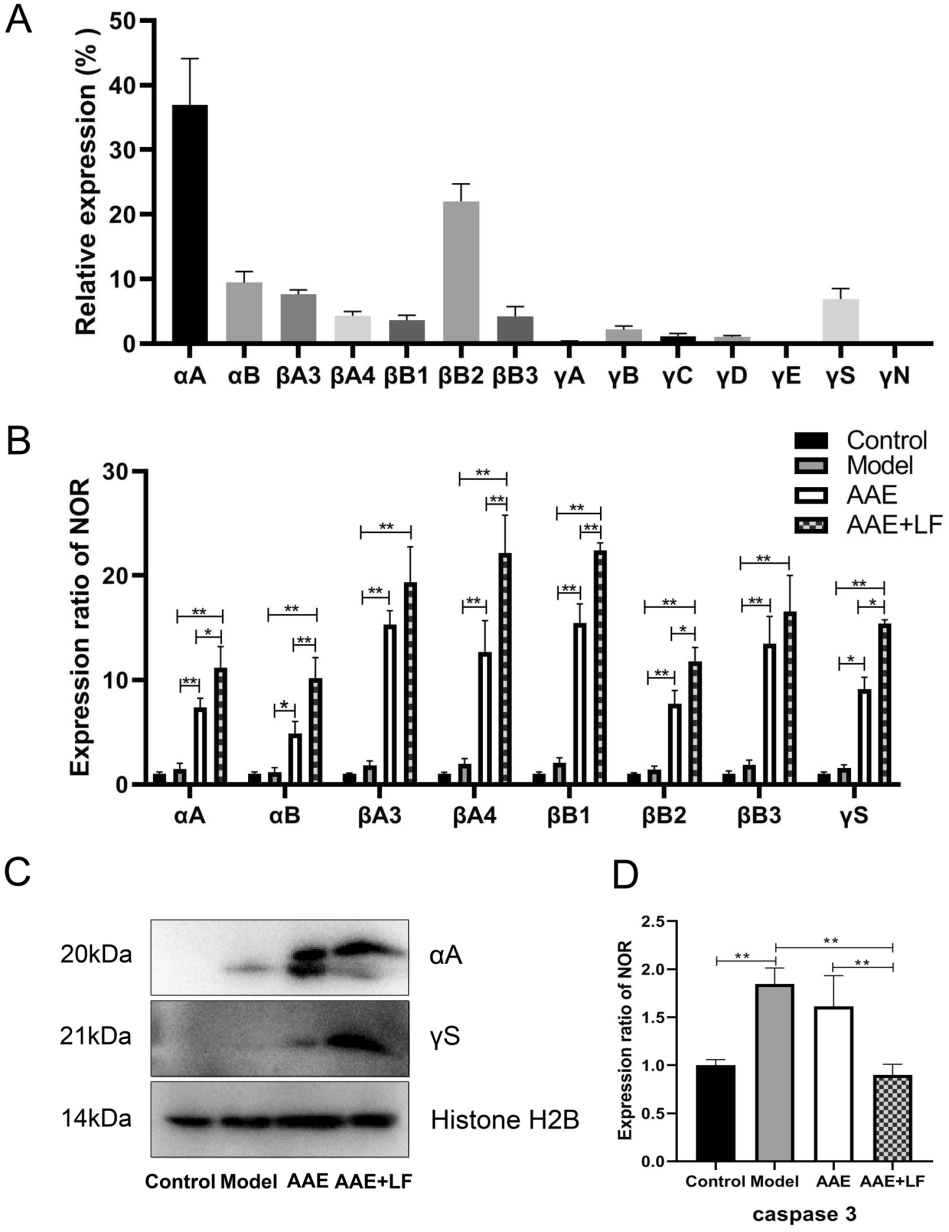
Expression of crystallin proteins and caspase 3 in the rat retina. (**A**) Relative expression percentage of different crystallins in the control rat (the mean amount of each protein expressed in NOR group was set to one or 100%); (**B**) Expression of α-crystallin A chain (αA), α-crystallin B chain (αB), β-crystallin A3 (βA3), β-crystallin A4 (βA4), β-crystallin B1 (βB1), β-crystallin B2 (βB2), β-crystallin B3 (βB3) and γ-crystallin S (γS) in different treatments, determined by mass spectrometry; (**C**) Immunoblotting of αA and γS in retina samples. Histone H2B detected as an internal reference to show a basic protein expression in each sample; (**D**) Expression of caspase 3 in different treatments. Data shown are the mean ± standard deviations (n = 3). *p < 0.05, **p < 0.01 (one-way ANOVA followed by Tukey’s test).

Previous study indicated that treatment with aronia fruit extract led to upregulation of the crystallin proteins in the stressed condition^13^. In this study, 14 crystallin proteins, including α-crystallin A chain (αA), α-crystallin B chain (αB), β-crystallin A3 (βA3), β-crystallin A4 (βA4), β-crystallin B1 (βB1),β-crystallin B2 (βB2), β-crystallin B3 (βB3) and γ-crystallin A-E (γA-E), γ-crystallin S (γS) and γ-crystallin N (γN) were found in rat retinas by mass spectrometry. As shown in Fig. 5A, the relative percentages of the above mentioned 14 proteins in total crystallin proteins of the control rat retinas were 37.00%, 9.42%, 7.64%, 4.29%, 3.61%, 22.01%, 4.25%, 0.33%, 2.20%, 1.17%, 1.01%, 0.01%, 6.91% and 0.15%, respectively.

The expressions of top 8 crystallins, including αA, αB, βA3, βA4, βB1, βB2, βB3 and γS were further compared among different treatments of rats. The 8 proteins expression had slightly increased 17–99% in Model compared with the Control, but statistically not significant (*p* > 0.05). However, the expressions of these protein were dramatically increased in both AAE and AAE + LF treatments. The expressions of αA, αB, βA3, βA4, βB1, βB2, βB3 and γS were 7.55-, 8.72-, 10.53-, 11.11-, 10.75-, 8.26-, 8.80- and 9.86-fold respectively (Fig. 5B) in AAE + LF, over those in Model. Compared with the AAE treated with anthocyanidin only, the treatment of AAE + LF significantly increased the expression of αA, αB, βA4, βB1, βB2 and γS by 51.69%, 110.10%, 74.19%, 45.34%, 52.58%, 68.39% (Fig. 5B), and tended to increase the expression of βA3 and βB3 by 26.60% and 22.95% (Fig. 5B), respectively. The results indicated that the combined treatment of AAE + LF led to a much higher upregulation of the protective crystallin proteins in the stressed condition. The similar result was also obtained in the immunoblotting analysis (Fig. 5C). Both αA and γS were not detected in the control rat retinas, and low expressed in the model. Their expressions were obviously increased in the AAE, and higher in the AAE + LF than in AAE treatment (see the Supplementary file).

In addition to the changes of crystallin proteins, the expression of caspase 3, a protein regulating the apoptosis, significantly increased in the Model compared with the Control (Fig. 5D). The elevation was slightly suppressed in the AAE treatment, but significantly inhibited by the treatment of AAE + LF. The caspase 3 expression in the AAE + LF group was reduced by 51.38% compared with the Model and by 44.34% compared with the AAE (Fig. 5D). The observations suggest a down-regulated apoptosis in rat retinas with the combined treatment AAE + LF.

### Effects of the treatments on the gut microbial composition of rats

**Figure 6:**
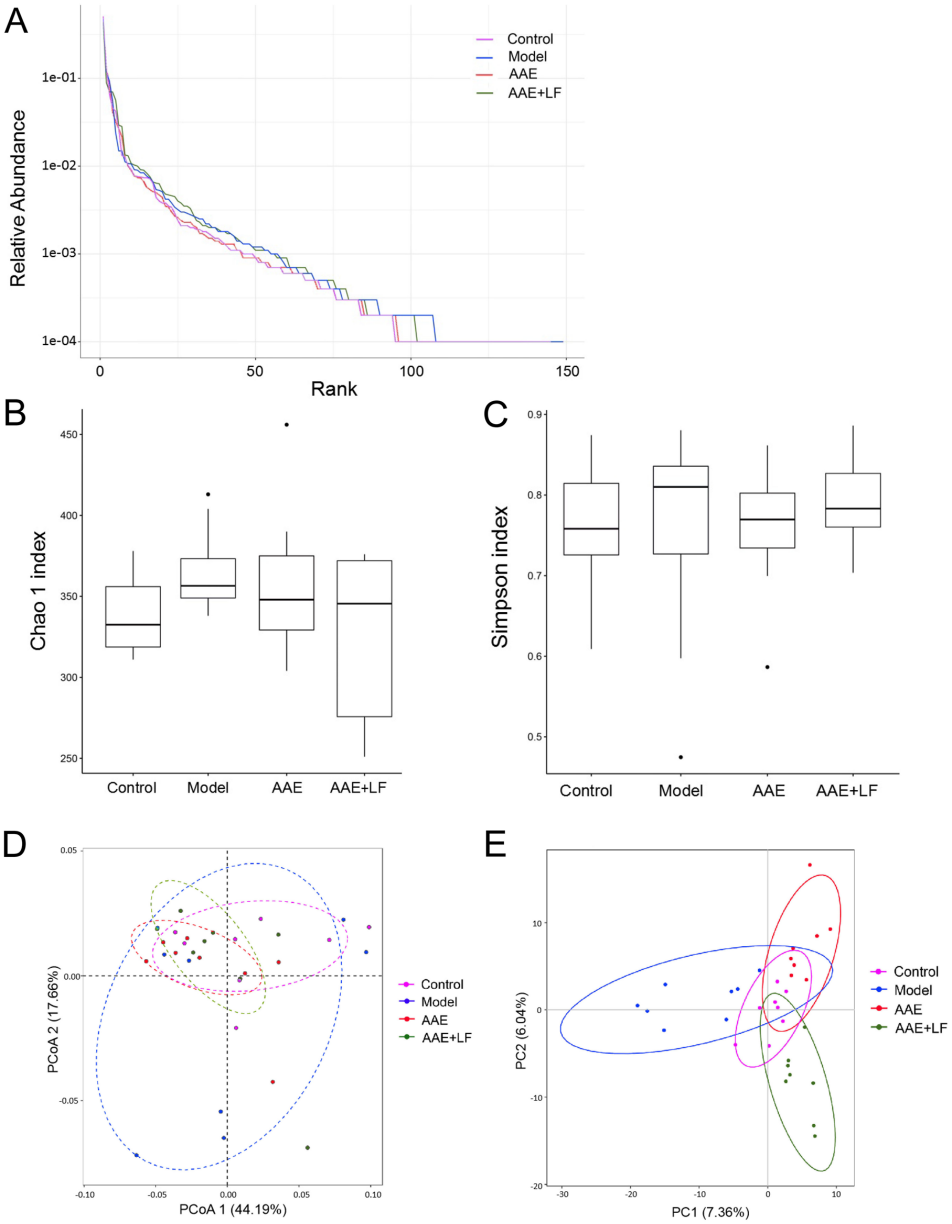
Gut microbiota structure patterns on genus level in the rat feces of different treatments. (**A**) Rank abundance curve of total microbial genera in feces of rats. The number of operational taxonomic units (OTUs) acts as a function of the number of sequence tags sampled; (**B**, **C**) Boxplots showing the α-diversity, including Chao and Simpson index in different treatments; (**D**, **E**) diagram showing the β-diversity, including the analysis results of Principal Components Analysis (PCoA) based on the weighted Unifrac distances of the gut microbiota and Partial Least Squares Discriminant Analysis (PLSDA) on genus level.

NaIO_3_-induced dry AMD was found to present a shift in the composition of gut microbiota. Compared with the control, the gut microbial richness and diversity tended to increase in NaIO_3_ damaged model, including Chao1 index increased by 7.22% and Simpson index increased by 8%. AAE or AAE + LF treatment rebuilt the gut microbial community, with the lower Chao1 and Simpson index compared with the Model and higher indices than the Control (Fig. 6A–C). PCoA and PLSDA revealed differences in microbial community structure with separated clusters between the Control and Model, while the structure in Control, AAE and AAE + LF treatments tended to be close (Fig. 6D,E). However, no indices exhibited statistical significance.

**Figure 7:**
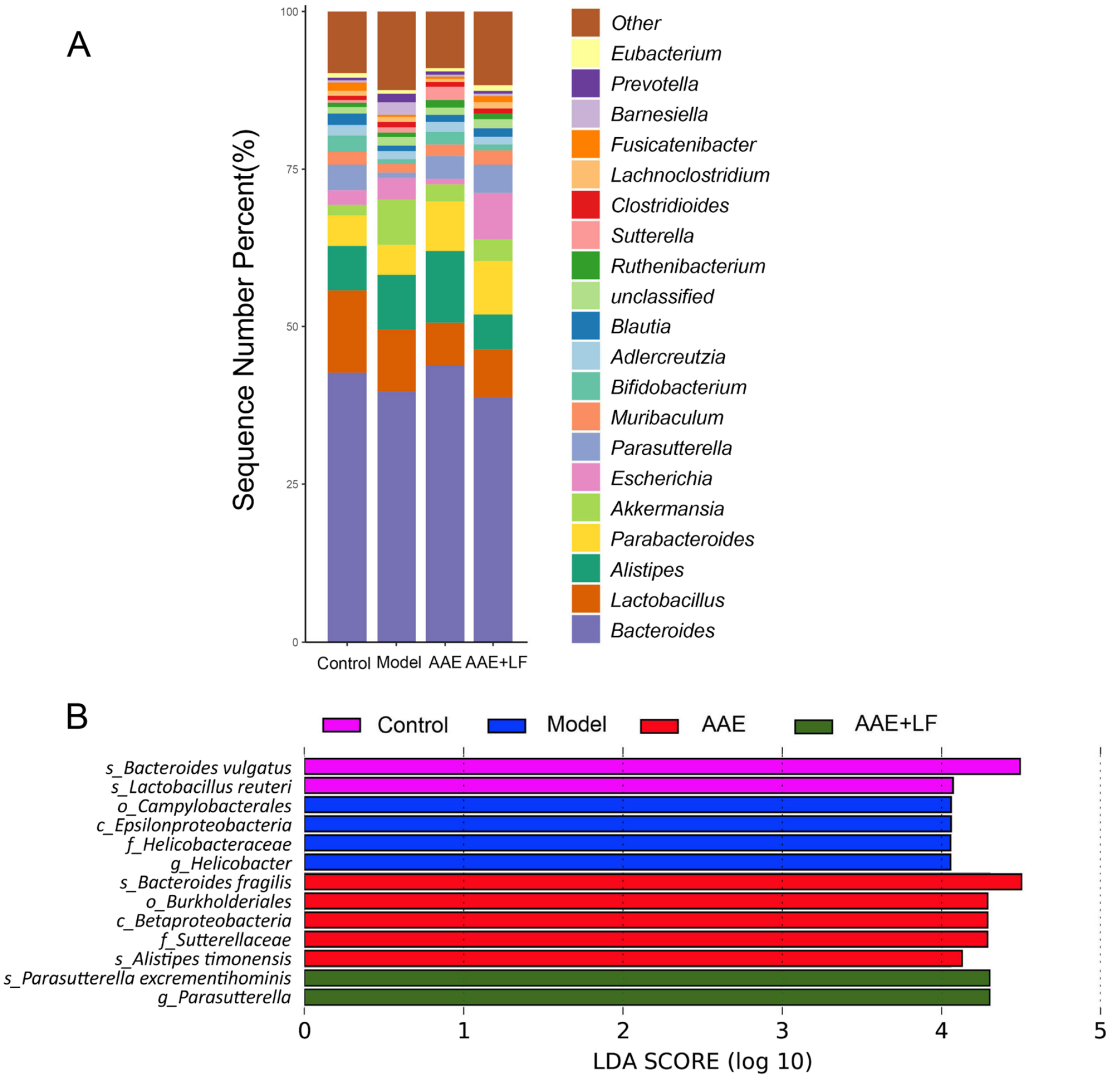
Alteration of gut microbiota following different treatments. (**A**) Relative abundance of the bacterial genera; (**B**) Linear discriminant analysis (LDA) effect size (LEfSe) was calculated to explore the taxa on genus level that more strongly discriminate among the different groups.

The relative abundances of the taxa on genus level were further compared (Fig. 7A). *Bacteroides* which accounted for about 40% of the total taxa represented the highest abundance, and *Lactobacillus*, *Alistipes*, *Parabacteroides*, *Akkermansia*, *Escherichia* and *Parasutterella* were the major taxa (above 3% of the mean abundance) in the 4 groups. There was no significant difference on the relative abundances of the major taxa among the different groups (Fig. 7A).

The metagenome analysis LEfSe approach was applied to identify the key phylotypes responsible for the difference on each level among the 4 groups. Members of the species *Bacteroides vulgatus* and *L. reuteri* in Control; the order *Campylobacterales*, class *Epsilonproteobacteria*, famlily *Helicobacteraceae*, the genus *Helicobacter* in Model; the species *B. fragilis* and *Alistipes timonensis*, the order *Burkholderiales*, the class *Betaproteobacteria* and the family *Sutterellaceae* in AAE; the species *Parasutterella excrementihominis* and the genus *Parasutterella* in AAE + LF were significantly prevalent than in the other groups that contributed to the difference of the gut microbiota following different treatments (Fig. 7B).

## Discussion

ERG responses can provide important clues in connection with the effect of the intervention on functioning of the retina^19^. In our results, the combined treatment of AAE together with LF significantly increased ERG a- and b-wave amplitudes and suppressed the cell layers disorder and ONL thinning, displayed a significant improvement on alleviation of rat retina damages compared with the anthocyanidins treated only. The results indicated that anthocyanidin combined with *L. fermentum* NS9 showed better protection on rat retinas from damages, both on rod and cone system, than aronia anthocyanidin treated only which was mainly function on cone system (Figs. 1 and 2).

The protective effects of the combined treatment (AAE + LF) on rat retina from NaIO_3_-induced oxidative stress were likely due to the increased antioxidant capacity and decreased apoptosis in retina. The up-regulated GPx activities and suppressed MDA production were observed in AAE treated group, but wider upregulation on antioxidant enzymes activities, including SOD, CAT, GPx, and stronger suppression on MDA level in the combined treatment AAE + LF (Fig. 4). Kim et al., also found paprika fermented with *L. plantarum* could mitigate the NaIO_3_-mediated reduction of SOD and glutathione (GSH) levels in the ocular tissues of mice and increase the protective effect of retinal degeneration^20^.

Oxidative stress promotes the formation of Aβ, which is caused by protein misfolding and aggregation^21^. Both αA and αB crystallins belong to the heat shock proteins (HSPs) family. They have high affinity with misfolded proteins to protect the RPE, photoreceptors and RGC from damages^17,22^. In our results, αA and αB crystallin expressions were upregulated 7.55- and 8.72-fold in the rat retinas treated with AAE + LF compared with the Model, and increased by 51.69% and 110.10% on the basis of AAE treatment (Fig. 5B). The high expression of αA and αB crystallin in retina may prevent oxidative stress-induced apoptosis of RPE and photoreceptors, which was supported by the down-regulation of caspase 3 (Fig. 5D).

In addition to αA and αB crystallins, βA3, βB2 and γS crystallin proteins have also been demonstrated to protect the RGCs from secondary degeneration^23^. Our results showed the AAE + LF combined treatment increased their expression by 26.60%, 52.58% and 68.39% compared with the AAE only (Fig. 5B). Earlier research identified that αB, βA1/3, βA4, βB2, and γS-crystallins existed in drusen, deposits basal to the RPE that may be associated with AMD^24^. In this study, we observed the expressions of αA, αB, βA3, βA4, βB1, βB2, βB3 and γS in AAE and AAE + LF groups were all significantly up-regulated compared with the Control and Model. Though the detailed mechanisms remain unclear, we believe the significant up-regulation of crystallins is important to protect the retina from stresses-induced antioxidant damage, cell apoptosis and loss of structure and function of the retina, which then postpone the development of dry AMD.

Compared with AAE treatment, AAE + LF displayed significant enhanced effects on the protection of rat retina, which suggests the function of *L. fermentum NS9* on the basis of AAE. Anthocyanidins are widely distributed in plant tissues where they mainly exist in form of glycosides or aglycones. *L. fermentum* has been reported to be beneficial to carbohydrate metabolism^25^. In our results, significant increase in the relative abundance of *Parasutterella* and *P. excrementihominis*, known as the saccharolytic strain, were detected following AAE + LF treatments. As a core microbiome member, *Parasutterella* is a high L-cysteine consumer, while L-cysteine plays great role in blood glucose regulation^26^. The proportion of *Parasutterella* was increased by carbohydrate consumption in rodent models^27^. *P. excrementihominis* have been reported to play an important role in maintaining host immunity^28^. Some probiotics can transform glycoside into aglycone to promote its absorption. Increased *Parasutterella* expression was found in ICR mice fed with flavonoid-enriched yogurt which was developed using *Lactiplantibacillus plantarum GY*^29^*.* In small intestine, anthocyanins are mainly absorbed as aglycones^30^. The improved expression of *Parasutterella* in AAE + LF group may enhance the bioavailability of anthocyanidins and improve immune function. Increasing research implicated that gut microbiota played important roles in many age-related degenerative diseases like AD and AMD^9,31,32^. Our study presented that *L. fermentum* NS9 combined with anthocyanidin extract could alleviate NaIO_3_-induced retinal damages, probably through the improvement of the retinal crystallins expression, antioxidant abilities, and microbiota dysbiosis. The combined treatment was significantly better than aronia anthocyanidin extract alone. Supplementing both with *L. fermentum* NS9 and anthocyanidin could be a promising way to prevent and alleviate retina degeneration.

## Methods

### Animals

Protocols used in this study have been reviewed and approved by the Animal Ethics Committee of the Institute of Medicinal Plant Development (No. SLXD-20201218031). All procedures were performed according to the Association for Research in Vision and Ophthalmology (ARVO) Statement for the use of Animals in Ophthalmic and Vision Research. All methods were performed in accordance with the relevant guidelines and regulations. In addition, all animal studies were conducted in accordance with ARRIVE guidelines. Forty male Sprague–Dawley (SD) rats 180–200 g were provided by the National Institutes for Food and Drug Control (Beijing, China, No. SCXK2017-0005). The animals were kept at 22 °C and in 12 h/12 h (7 AM to 7 PM) light/dark cycle.

### *Lactobacillus fermentum* NS9 and aronia anthocyanidin extract

The *L. fermentum NS9* strain was inoculated into MRS (De Man, Rogosa and Sharpe agar) medium at 37 °C for 12 h. The bacteria were collected by centrifugation at 3000 rpm for 5 min and washed twice with phosphate buffer saline (PBS, pH 7.4). The strain was resuspended at a concentration of 10^8^ colony-forming units (CFU)/ml.

The aronia anthocyanidin extract was the purplish red powder of water extract from *Aronia melanocarpa* fruits, purchased from the Greater Hinggan Gebei Frigid Zone Biotechnology Co., LTD (Heilongjiang, China). The powder contains 10% starch (exogenously added during preparation of the powder), 10.3% anthocyanidin, and other water-soluble nutrients including saccharides, proteins, and dietary fiber from *Aronia melanocarpa* fruits.

### Treatments of the rats

The rats were randomly separated into Control, Model, AAE and AAE + LF groups. In AAE group, aronia anthocyanidin extract at 600 mg/kg body weight (anthocyanidin at 60 mg/kg body weight) in distilled water was administrated orally once a day for 28 days. In AAE + LF group, the rats were treated with 60 mg/kg body weight AAE and 10^8^ CFU/ml *L. fermentum* NS9 per day for 28 days. In the Control and Model groups, rats were orally administrated with distilled water. The murine NaIO_3_ injection model is a widely used AMD model^16–18^ and we had confirmed 30 mg/kg body weight as an appropriate dose in our preliminary experiment. A single treatment of NaIO_3_ at 30 mg/kg body weight was intravenous injected in the Model, AAE and AAE + LF groups on the 8th day.

### Electroretinographic analysis

The full-field ERG of rats were recorded using an ERG recording system (D430 Diagnosis, USA) as previously reported^13,33^. Before the measurement of ERG, the rats were dark-adapted for 12 h. Twenty minutes before the recording, the animals were anesthetized by intramuscular injection with the mixture of ketamine hydrochloride and xylazine hydrochloride at the dosages of 100 mg/kg and 15 mg/kg, respectively. Eye drops containing 0.5% tropicamide and 0.5% phenylephrine hydrochloride were administered to the eyes of rats to dilate the pupils. Both a- and b-waves amplitudes were recorded and statistically analyzed.

### Histologic analysis of the rat retina structure

Both eyes were removed after the rats were euthanized by intramuscular injection of ketamine hydrochloride associated with xylazine hydrochloride. The retina were fixed and stained with H&E as previously reported^34,35^. For each section, digitized images of the entire retina were taken with a digital camera (Leica DMi8, Wetzlar, Germany) at 200 × magnification. The thickness of the outer nuclear layer (ONL) was measured with Image J software (US National Institutes of Health, Bethesda, USA). Twelve locations for each retinal section were measured, starting from either side of the optic nerve, with each segment 0.5 mm apart. The 12 measurements were averaged as the mean ONL thickness.

### Evaluation of antioxidant capacity of rat retina

The retina samples taken from the eyeballs of rats were homogenized with phosphate buffer saline (PBS, pH 7.4) and centrifuged at 3500 rpm for 10 min, and the supernatant was collected. The activities of SOD, CAT, GPx and the content of MDA in the supernatant of each sample were determined spectrophotometrically using the measuring kit (Nanjing Jiancheng Bioengineering Institute, Nanjing, China).

### Mass spectrometric analysis of retina proteins

Rat retina proteins were identified and analyzed using tandem MS following the previous protocol^13^. The retina tissues were lysed by sonication in 8 M urea buffer. After digested with trypsin, the peptides were analyzed on an Orbitrap Q Ex-active HF mass spectrometer coupled with an online EASY-nLC 1200 nano-high-performance liquid chromatography (HPLC) system (Thermo Fisher Scientific, USA). The mass spectrometry results were analyzed and quantified using PEAKS Studio (Waterloo, Canada).

### Immunoblotting

The immunoblotting analysis was performed as previous reported^13,36^. The membrane with retina proteins was washed with 5% skimmed milk in Tween/Tris-buffered saline (TBST) to block nonspecific binding and then incubated with primary antibodies against α-crystallin A chain and γ-crystallin S. Immunoblots were performed using SuperSignal Western Pick Plus (#34577, Thermo Scientific) . The blots were cut prior to hybridisation with antibodies during blotting.

### Metagenome analysis of gut microbiota

The fecal specimens of rats were obtained at the end of rat treatments in Section “Effect of the treatments on the expression of crystallin proteins and caspase 3 in rat retina” and immediately stored at – 80 °C until analysis. Microbial DNA extraction and metagenome analysis were conducted by Microeco Tech Co., Ltd. (Guangdong, China) as previously reported^37^.

For bioinformatic analysis of microbiome sequences, Kraken2 (v2.0.7) was employed to assign reads to taxonomy and Bracken (v2.5.0) was used to accurately estimate taxonomic abundance. LEfSe analysis was applied to identify differentially abundant bacterial taxa among groups. Only those taxa that obtained a log linear discriminant analysis (LDA) score > 4 were ultimately considered. To determine the false discovery rate (FDR), the multiple test correction method, Benjamini–Hochberg was used.

### Statistical analysis

Results are presented as the mean ± standard deviation. Differences between groups were assessed by one-way ANOVA, followed by Tukey’s test. *p* < 0.05 was considered statistically significant. Statistical analyses were performed using Prism 8.0 (GraphPad Software, San Diego, CA, USA).

## Supplementary Information

Supplementary Figures.

## Data Availability

The datasets generated during and/or analyzed during the current study are available from the corresponding author on reasonable request.
