# Supplementary material for: Combination of Lactobacillus fermentum NS9 and aronia anthocyanidin extract alleviates sodium iodate-induced retina degeneration
Source: Sci Rep. 2023 May 24;13:8380. doi: 10.1038/s41598-023-34219-3 (PMC10209211; doi:10.1038/s41598-023-34219-3)
Supplement: Supplementary file 1 — Supplementary Figures. [file 41598_2023_34219_MOESM1_ESM.docx]

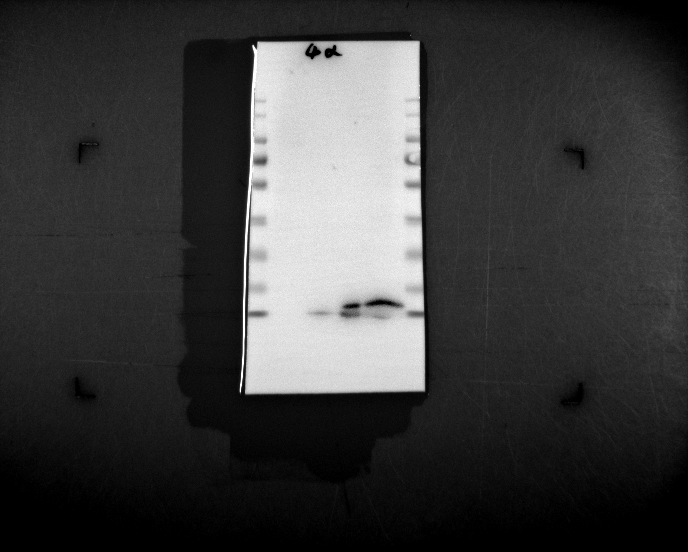

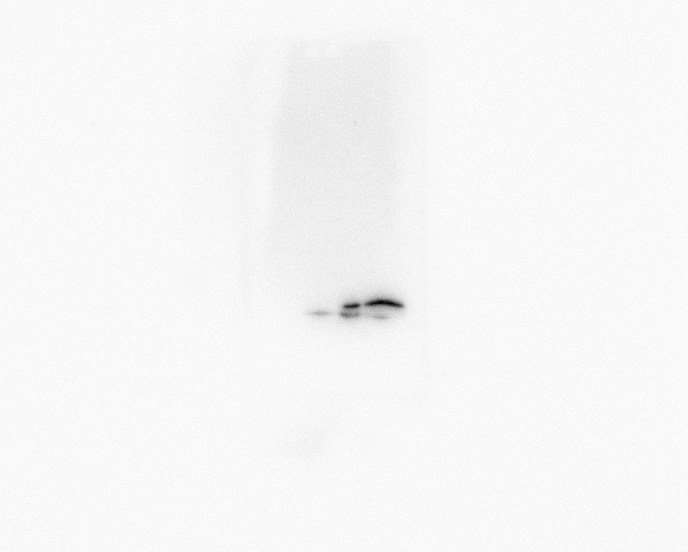

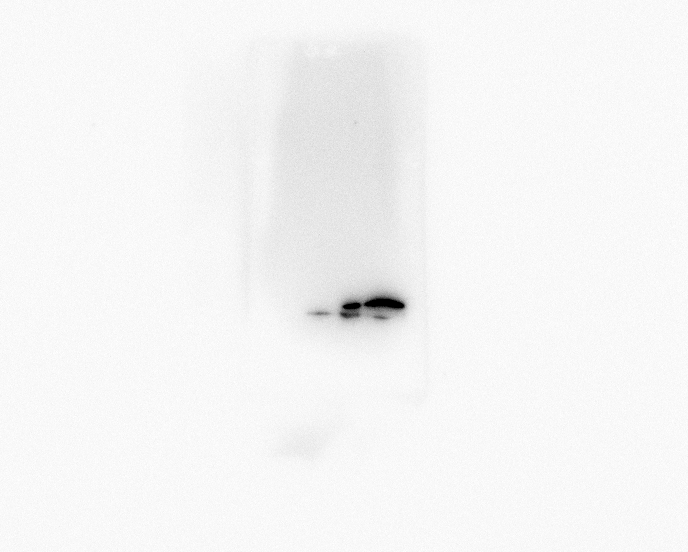


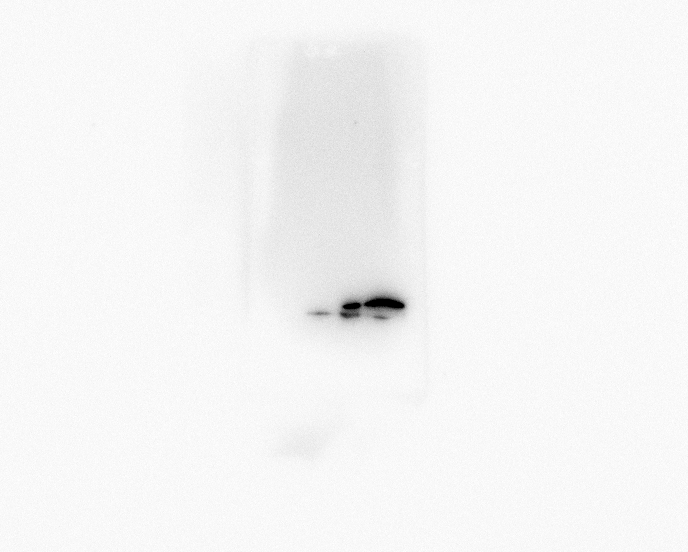


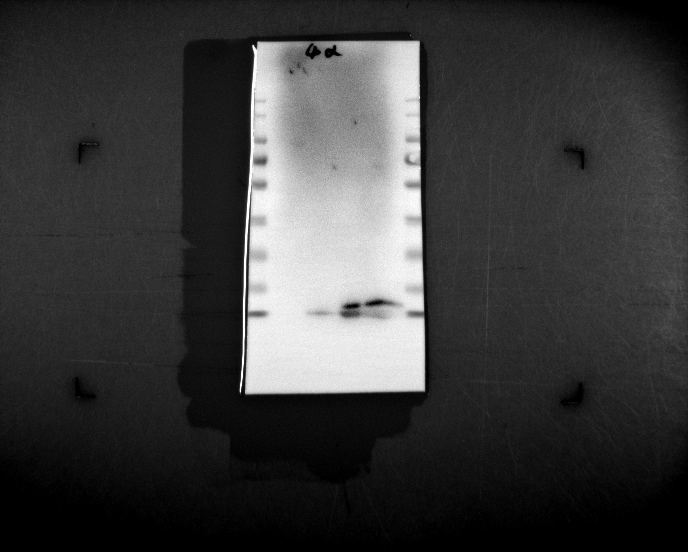

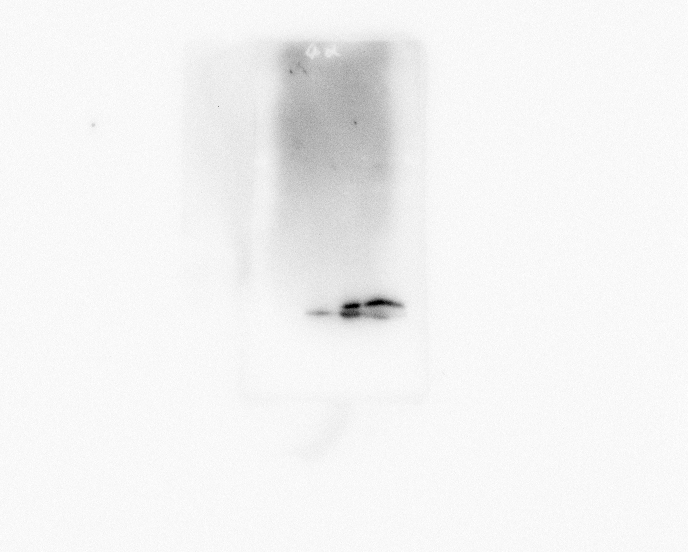


Figure S1 The original images of α-crystallin A

**
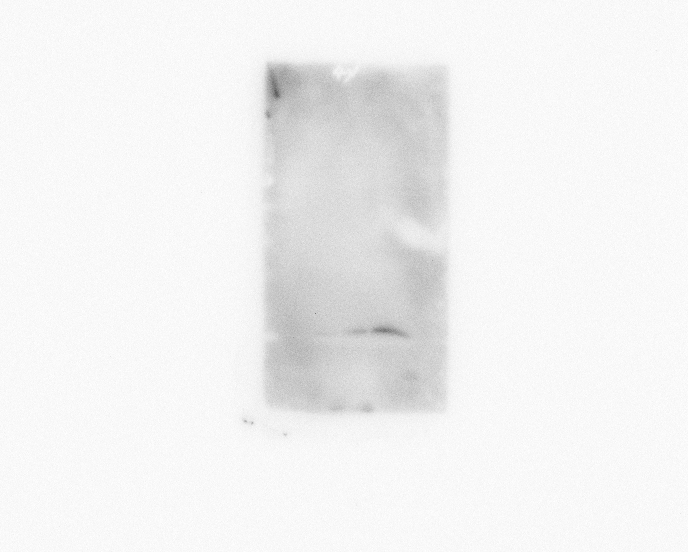

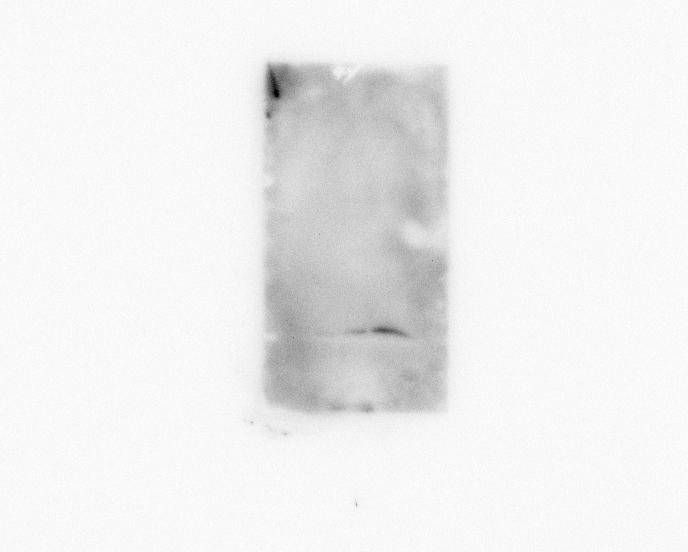

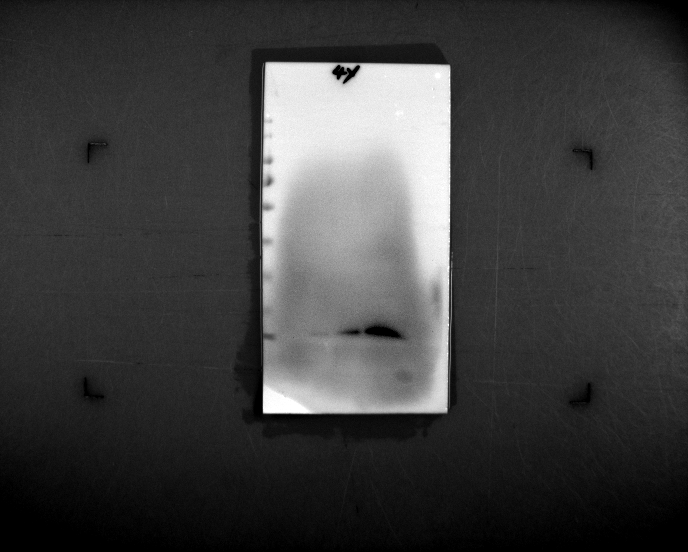
**

**
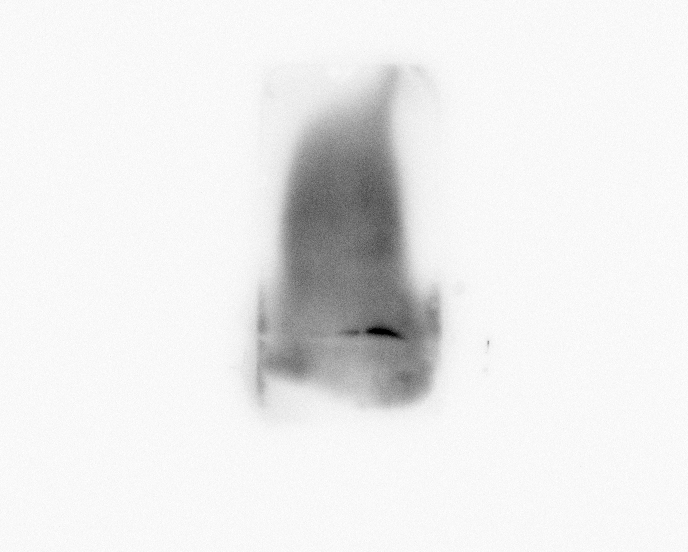

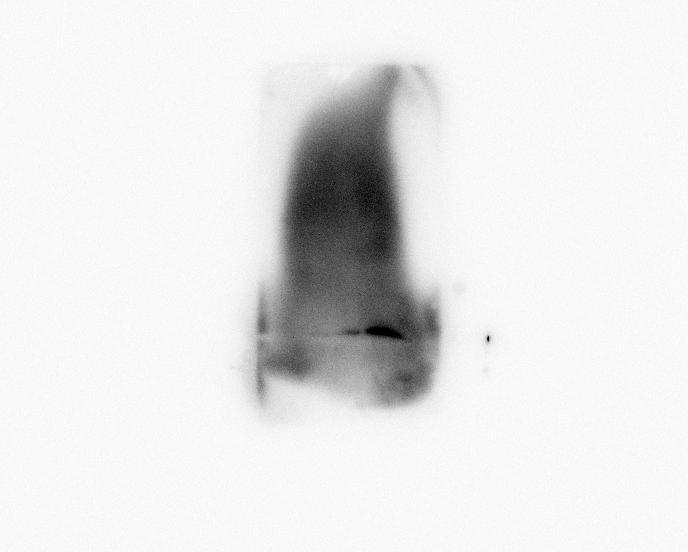

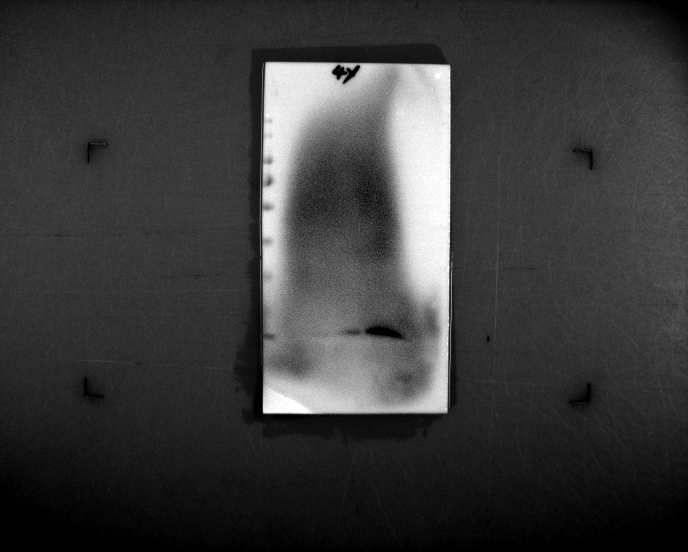
**

Figure S2 The original images of γ-crystallin S


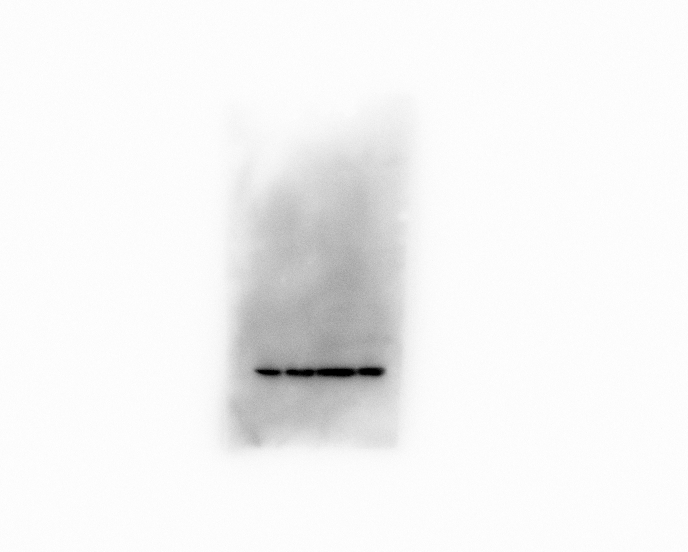

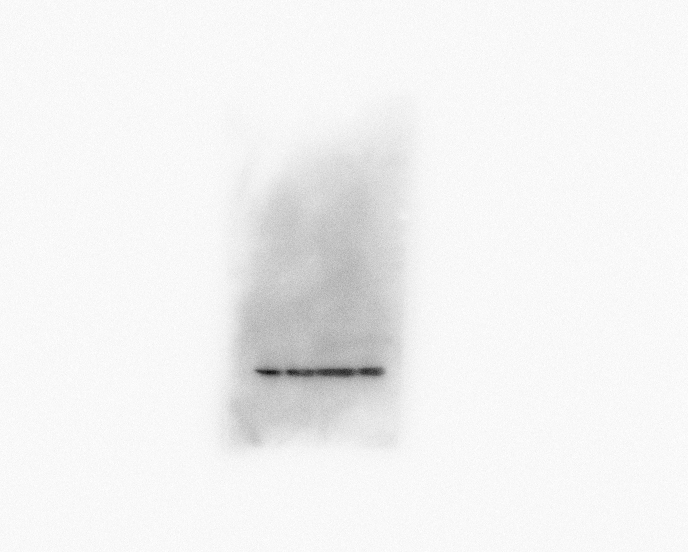


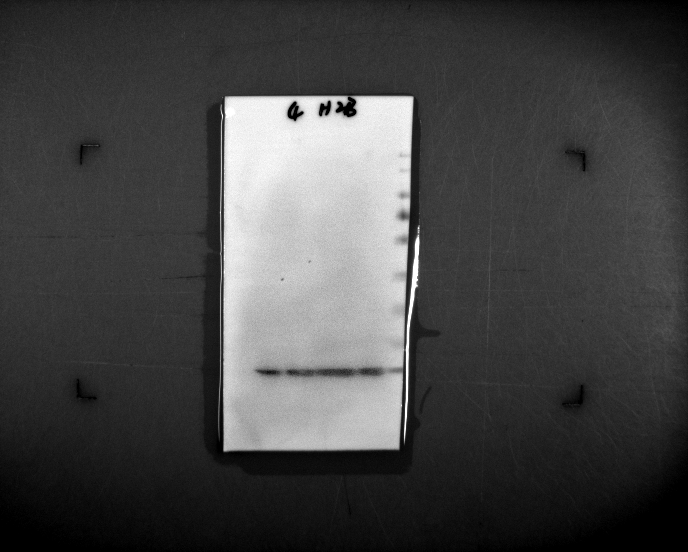

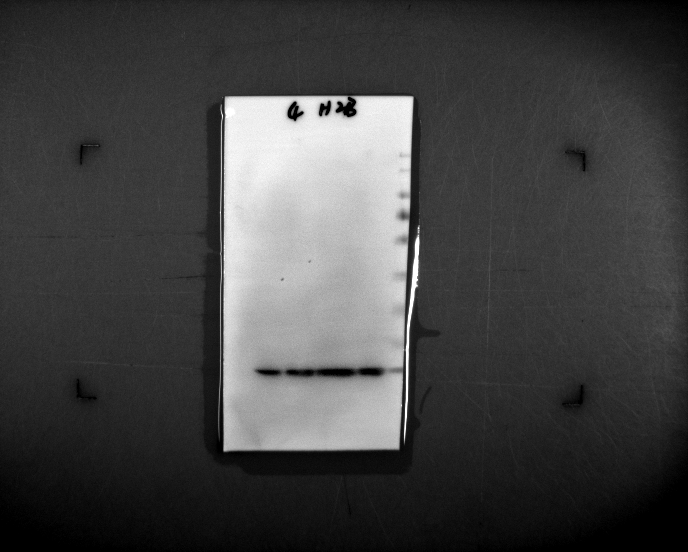


Figure S3 The original images of Histone H2B


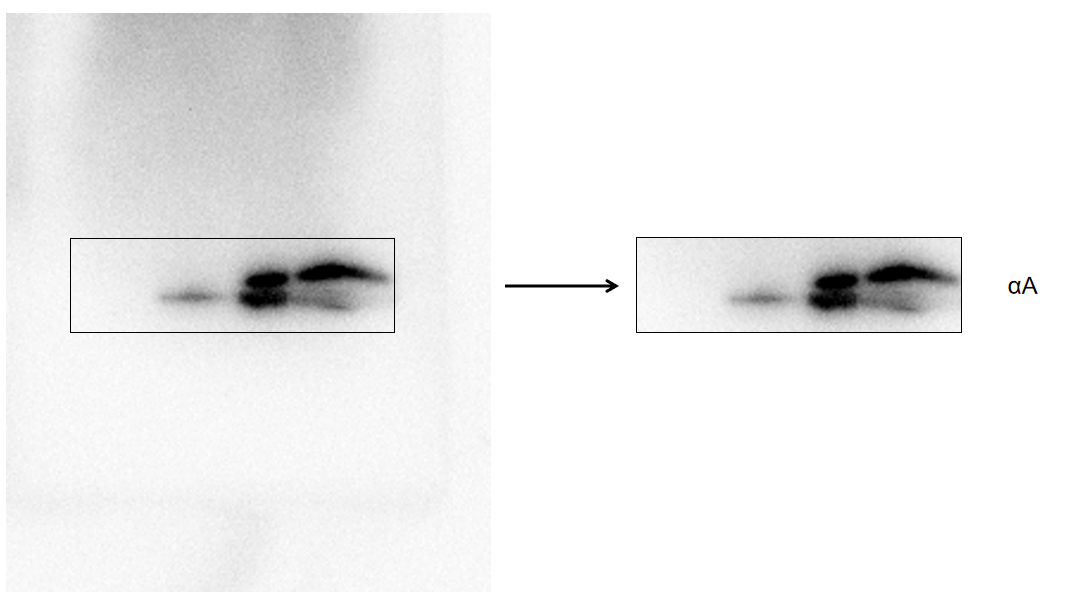


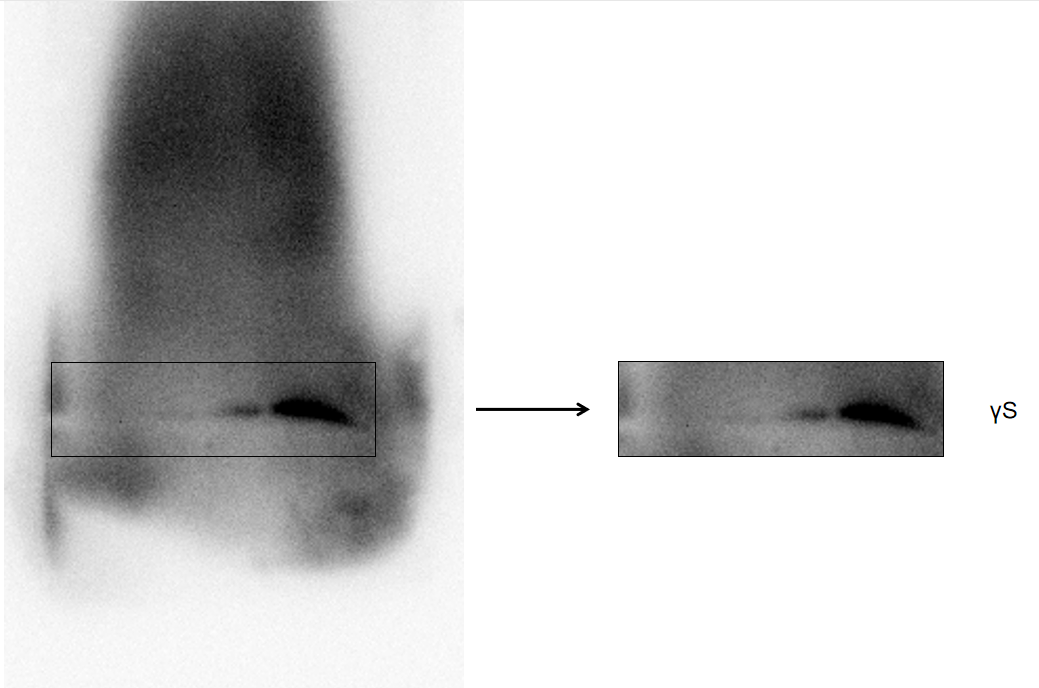

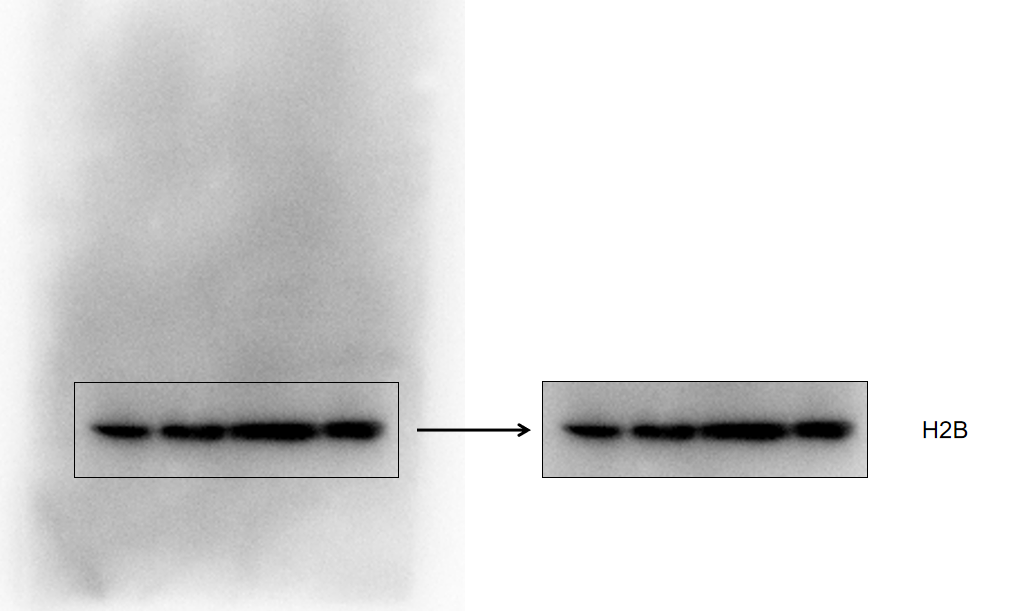


Figure S4 The images in the manuscript were cut from the original images


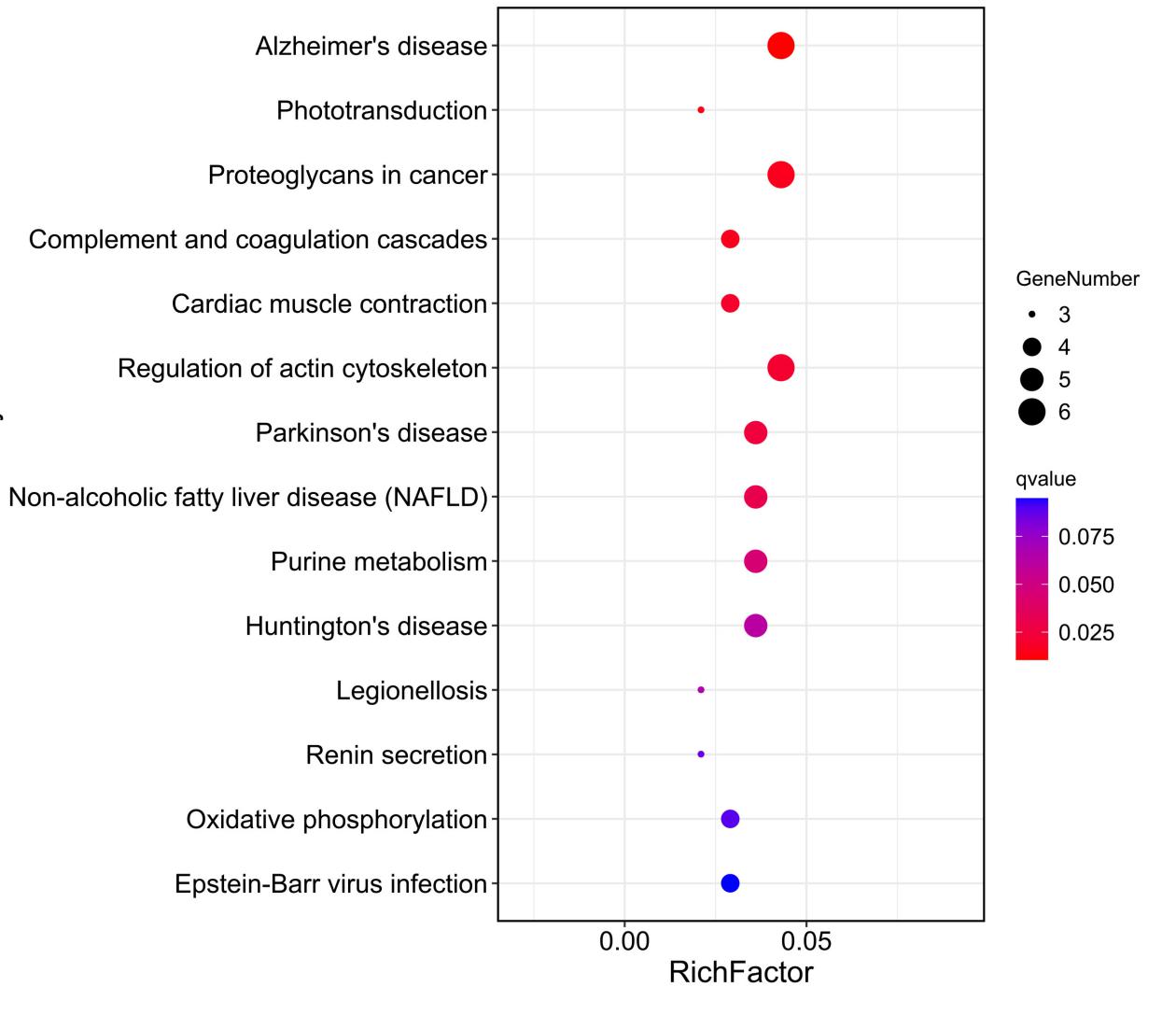


Figure S5 KEGG analysis of differential proteins between the AAE+LF and Model

Statistics of enriched KEGG terms display 141 differentially expressed retinal proteins between AAE+LF and the Model. The KEGG analysis was performed as previously reported^[1-3]^ (URL: [www.kegg.jp/kegg/kegg1.html](http://www.kegg.jp/kegg/kegg1.html)). The size of the point indicates the number of differentially expressed proteins in this pathway, and the color of the points corresponds to a different q-value range. R scripts were used for data analyses and visualizations. Differentially expressed proteins between groups were identified by a two-tailed *t* test. To determine the false discovery rate (FDR), the multiple test correction method, Benjamini-Hochberg was used. The criterion to identify significant differences was Fold Change (FC)>2 and adjusted *p* value (q value) <0.05.

References:

1. Kanehisa, M. and Goto, S. KEGG: Kyoto Encyclopedia of Genes and Genomes. Nucleic Acids Res. 2000;28(1):27-30 . [PMID:10592173]
2. Kanehisa, M. Toward understanding the origin and evolution of cellular organisms. Protein Sci. 2019;28(11):1947-1951. [PMID:31441146]
3. Kanehisa, M., Furumichi, M., Sato, Y., Kawashima, M. and Ishiguro-Watanabe, M. KEGG for taxonomy-based analysis of pathways and genomes. Nucleic Acids Res. 2023;51(D1):D587-D592. [PMID:36300620]
